# Parliament and the Professions

**Published:** 1921-07-09

**Authors:** 


### Parliament and the Professions.

### Deaths Due to Vaccination.

The Minister of Health was asked by Mr. Waterson whether letters of inquiry are still addressed to medical men who certify deaths as due to vaccination. Sir A. Mond replied that twenty such letters have been sent during the past ten years. It is also the practice in these cases for a medical officer of the Ministry to make personal inquiries into the circumstances.

### Leave in the Indian Medical Service.

In answer to a question put to the Secretary of State for India, it was stated that owing to conditions created by the war it has not been possible to grant leave to the Indian Medical Service to the extent desirable. Every effort is being made to bring the Service up to full strength, and so make it possible to grant leave more freely. In the meantime the best that can be done is to restrict the amount of leave that may be taken by an officer at one time, so that as many as possible may come home.

